# Professionalism vs. Commercialism*Extracts from an address to the Missouri Dental Association in Kansas City, and printed in the *Dental Cosmos*. September, 1920.

**Published:** 1920-10

**Authors:** C. N. Johnson


### PROFESSIONALISM vs. COMMERCIALISM *

BY C. N. JOHNSON, D.D.S.

Commercialism has many forms. I am going to point out some as I see them, and I wish you would take my strictures as coming, not from a man disgruntled or unduly exercised, but from a man intensely interested in the reputation of this noble profession of ours.

As I view it, most of the commercialism of to-day has its manifestations in an exploitation of the people on the part of some dentists. That exploitation presents itself in many guises. I will give one or two instances of what has come under my own observation as illustrative of what is going on in many localities all over this country.

One case was a patient of mine who upon her marriage moved to another city. When visiting in Chicago she was in the habit of calling on me to have her mouth looked over. Some time ago her baby was born, and for awhile she was unable to travel and an accident to one of her teeth required a visit to a local dentist in her city of residence. When this dentist examined her mouth he advised her to have an X-ray taken of all her teeth. There was no occasion for such advice, because she had no pulpless teeth and there was absolutely no pathological condition present. After an examination of her teeth he made much

* Extracts from an address to the Missouri Dental Association in Kansas City, and printed in the *Dental Cosmos*, September, 1920.

ado about the condition he claimed to find, and said to her, "You must come here every day for two months to have your mouth put in decent condition." This alarmed her very much, and she had her husband write to me to ask if I would care for her if she came to Chicago. She came, and the actual fact is that it took only five short sittings to put her mouth in the best possible condition. All this in the face of the dentist's assurance that it would take two months to complete the work. Do you know why he gave her that advice? Because that woman happened to have some money. That is not professionalism; it is commercialism of the basest sort.

Another instance where a man's teeth were ordered out. This man came to me on the recommendation of one of my patients. I looked the mouth over carefully and said, "I do not agree with that diagnosis." There was a time when I was inclined to be diplomatic with my fellow practitioners and to shield them, but I have now reached a point where I am rather blunt in stating my views to the patient or to the one who refers that patient to me. The physician had sent this patient to the X-ray man, and the X-ray man handed him a diagnosis with which he returned to the physician; he was then referred to a dentist and the dentist ordered the extraction of all the teeth, basing his advice on the X-ray diagnosis. The patient was frightened, naturally, and came to me. I said frankly that I did not agree with the diagnosis. He said, "What shall I do?" I replied, "Tell your physician to call me up." The physician did so and said to me, "It has occurred to me it would be a good thing if that man had his teeth all out, because I doubt whether he will take care of them." I asked, "Do you mean to tell me a man who says he would rather lose his left hand than his teeth won't take care of them?" He said, "Did the patient say that?" I told him he did. Just before the New Orleans meeting I received a letter from the patient to this effect: "I have had my tonsils taken out and have just had a culture made from my saliva, and they are going to see if there is any streptococcus there, and if there is, I will let you know." I took the time, even though I was in a hurry to leave for New Orleans, and wrote that man, saying, "My dear sir, I predict that they will find the streptococcus there because they can demonstrate it in every mouth." Finally he came back to my office to insist upon becoming my patient.

The point I want to make is this: A man who knew enough about bacteria to look for streptococci knew that he would find them in that mouth. That is exploiting the people and frightening them unnecessarily. This man I referred to still has all of his teeth and he is in splendid physical condition.

I make no complaint whatever about the investigator who looks for the truth and tells his patients what he finds, but I am quarreling unceasingly with the practitioner who takes advantage of his professional status to make an impression upon a patient and leads him to believe his condition is serious when it is not.

Then some of the courses in the curricula of the schools on business building lay too much stress upon the necessity of higher fees. The custom of taking money from the people without a consideration of the patient's point of view is not right, ethically or morally. There is too much of that going on to-day all over the country.

### EXPLOITATION OF THE RADIOGRAPH

Now, a few words about the value of the X-ray. I wish first of all to pay my tribute to the usefulness of this wonderful process. There is nothing that has come into our profession, in my judgment, that is so valuable in the hands of a conscientious and able investigator. I take off my hat to the radiodontist who is sincere and earnest and equips himself properly to search for the truth, and I wish to pay my tribute to the practitioner who sincerely endeavors to apply the findings of the radiograph, but the way this thing has been exploited to-day is a disgrace to our civilization. I know how it works out with us in Chicago; I do not know just what the situation has been in St. Louis and Kansas City; but the method of procedure is something like this: A patient becomes ill. He goes to his physician, and this being the era of attention to the condition of the teeth in the medical fraternity, he is at once sent, without adequate physical examination, to the radiodontist to have an X-ray made of the teeth. Now, the thing the radiodontist does not do in his investigation could be told in a few minutes; the things he does do can never be told. The radiodontist, whether he is a qualified dental practitioner or a physician, or whether he is a man who simply takes pictures and does not know anything about the anatomy of the mouth or etiology of disease and does not know anything about pathology, will write a diagnosis of that case. He will send it to the physician, or, what is worse, hand it to the patient. The things he writes upon that diagnosis in many cases are a disgrace to humanity to-day.

He will write "abscess," "infection," and if he can not find anything else, he will write "pyorrhea," and now they have adopted that term "osteoclasia," which makes a most wonderful impression upon the patient and usually succeeds in scaring him nearly to death. The physician, who in many instances knows nothing about the etiology of the teeth, takes that radiograph and refers the case to the exodontist with his recommendation to extract. The family dentist is too frequently ignored. He has records of all these operations and can throw some light on them, and in all conscience has as much interest in the patient's welfare as the radiodontist. The impression made upon the minds of the patients reading such terms as "abscess" and "infection" is difficult to eliminate. People have died of infection, as they know. Abscess is a dangerous condition, and the worst feature of all is that these men will find infection in every case. It does not have to be there in order to be found. One of our leading X-ray men has said that it was a brave radiodontist who had the courage to say that he did not find anything pathological. I have not met many men of that type in my experience in Chicago. I hope there is such a man in Missouri. I at least know that there are many men in Missouri who are doing careful and conscientious X-ray work and I pay my tribute to them tonight. They show us things we can not find ourselves. But my understanding of the position of the X-ray man who acts as an intermediary in these investigations is that he shall not give a diagnosis to the patient. I will not allow one to be handed to one of my patients if I can help it. No radiodontist, I care not how expert he is, has a right to say, "You have an abscess at the apex of that tooth," because he can not demonstrate an abscess by the X-ray. The best X-ray men know that and they are frank enough to tell you, and I want to say here that the future of the benefit of the X-ray lies with the men of that type, who are conservative enough to be constructive instead of destructive.

I had a case just last week of the wife of a physician, who came to my office with an X-ray diagnosis reading in the usual way, as described above. This diagnosis had been made by an X-ray man who was also a dentist; she was referred back to her own dentist. The consensus of opinion of those two men was that the lower right first, second, and third molars and bicuspids should be extracted. The pulps were alive in all of those teeth except the first bicuspid. On that X-ray diagnosis was written "Serious infection at the apex of the third molar"—and it was a tooth with a live pulp! On what ground did he make that diagnosis? Dr. Simpson of

St. Louis can tell you. The inferior dental canal runs along there and it cast a shadow. The radiodontist called it an infection. God save the people!

That woman was very much alarmed; her husband being a physician, she knew the consequences of infection. I told her to go back to her husband and tell him that those teeth did not have to come out, and to tell her dentist they did not have to come out, and if there was any further controversy about it to call me up on the telephone. The dentist never called me up, which was rather lucky for both of us perhaps. The husband called me up and said it had greatly relieved his mind. That is another case of exploitation of the people which I am contending against. Let me tell you the X-ray man must stop giving a diagnosis to patients and frightening them unnecessarily. Let him tell all he knows or thinks he knows or can interpret to the physician or to the patient's dentist, but with the explicit understanding that the information does not go to the patient unless it is necessary. Very many patients have been stampeded with fright into the extraction of numberless perfectly useful and harmless teeth, and it is time to bring that sort of thing to a halt.

### CURETTING SOCKETS AFTER EXTRACTION

Another thing that has broken out in the profession is this wonderful idea of curetting every cavity or socket from which a tooth has been extracted. For years we went along extracting teeth, sometimes not extracting as many as we should, but extracting teeth that were badly diseased, and the gums healed up promptly and the patient got well. And now all at once we have discovered those sockets must be cureted. Why? Many of these curetting operations are done to see how deeply the operator can dig into the purse of the patient. Curetting is sometimes necessary, but the cases are very rare relatively to the number of teeth that are extracted. A professional man is entitled to a good fee for his services, if he works conscientiously and carefully. His years of usefulness are limited and he should be recompensed to a degree that he is not compelled to become dependent upon charity in his old age; but the exaltation of the idea of fees before service is pernicious, and must be combated. A man in Chicago the other day made the statement that unless ninety per cent. of your patients complain about their bills your fees are not high enough. I should say that if nine per cent. complained there was something wrong with a man's methods.

The professional man should have the question of fees come up in his practice very seldom after he has been in a community a few years. Let him get good fees, but this constant contention over fees is a demoralizing habit in a profession.

I have given the best energy of my life in the last few years without expectation of a fee at all. It has come about in this way. Some of the medical men in Chicago have heard that I combated this universal extraction of teeth, and have gotten in the habit of sending patients to me with X-ray diagnosis. The patient usually comes with a diagnosis from the X-ray man that there is grave infection there. I look them over as conscientiously as I can and send them back to their dentist with the statement: "Tell your dentist frankly what I think about the case, and have him call me up and we will consult about it." Now that takes up time, and it would be perfectly legitimate to charge a fee, but I can not bring myself to do it. Do you know why? That patient not feeling well goes to the physician; he is sent by the physician to the radiodontist, and they both charge him a fee; then he comes to me and I send him back to his dentist, who expects another fee, and if I make the chain complete by charging a fee, after the patients go through this process they begin to think it is nothing but graft. I want to do something in my profession, if I can, to do away with that idea. I do not want the profession to have the reputation of grafting and I make all sorts of excuses for not giving a bill to the patient. My own office force blame me for not making out a bill, the physician blames me, but I am going to have a clear conscience in the matter. I do not tell the patients that I do it for them. I would not do a thing as demoralizing as that, because I want them to think that professional service is worthy of a fee, but I say, "I am doing this for your physician, or for your dentist as a matter of courtesy," and I get them out of the office with the feeling that the chain of graft is at least broken in one part. As I say, I have given the best energy of my life to that and I am going to continue to do it.

When the time comes that dentists think more of the money they make than of the service they render, when they expend their energy in exploiting the people rather than in conserving their welfare, when they study methods of business more than methods of practice, when they exalt the god of mammon above the divinity of service, when the sordid and selfish shall have assumed ascendency over the sublimity of sacrifice, then this profession of ours shall sink in the deepest depth of degradation, and we shall be known as men not fit for professional status, not worthy to associate with those of culture and refinement; not even entitled to a place beside the common daily laborer, because we have besmirched our profession, and betrayed a trust reposed in us by the sturdy old pioneers of the days gone by, who by their unselfish sacrifice and devotion to humanity have handed down to us a profession imbued with high ideals, whereby we are enjoined to serve humanity in an honorable way rather than to use our calling as a cloak to impose upon the people.

Gentlemen, it remains for you and for me to choose whether we shall exalt commercialism above professionalism, or whether we shall so conduct our affairs that while reaping a legitimate material reward we shall at the same time as professional practitioners be able to look every man in the face, and not hang our heads in shame.

